# Entrectinib can induce nerve cell damage by inhibiting PI3K-AKT and TGF-β signaling pathways

**DOI:** 10.3389/fphar.2025.1489210

**Published:** 2025-02-13

**Authors:** Qingshan Tang, Jiachen Dong, Feng Zhang, Dan Zhao, Qi Yang, Jiayu Wen, Yuhao Sun, Jifu Wei, Zhixian Liu

**Affiliations:** ^1^ Jiangsu Key Laboratory, Pharmacology and Safety Evaluation of Chinese Materia Medica, Nanjing University of Chinese Medicine, Nanjing, China; ^2^ Jiangsu Cancer Hospital, The Affiliated Cancer Hospital of Nanjing Medical University, Jiangsu Institute of Cancer Research, Nanjing, China

**Keywords:** entrectinib, nerve cell damage, THBS1, Pi3k-akt, TGF-β

## Abstract

**Background:**

The tyrosine receptor kinase inhibitor (TRKi) entrectinib is used to treat neurotrophic tyrosine receptor kinase (NTRK) fusion-positive solid tumors and ROS1-positive patients. Despite its impressive efficacy against cancer, the clinical application is still limited by the central nervous system (CNS)-related toxicities. However, the precise mechanism of such CNS-related toxicities remains elusive.

**Methods:**

The effect of entrectinib-induced nerve cell damage was evaluated by the nerve cells (PC12, HT22 and SK-N-SH) based *in vitro* models. Various assays, including CCK-8, colony formation and EdU incorporation assays were utilized to estimate the cellular viability and proliferation ability. Cell apoptosis was measured by flow cytometry. Next, transcriptome sequencing technology was performed to identify differentially expressed genes (DEGs). Gene ontology (GO), kyoto encyclopedia of genes and genomes (KEGG) analysis and gene set enrichment analysis (GSEA) were applied to predict the potential functions of DEGs. Quantitative real time polymerase chain reaction (qRT-PCR) and Western blotting assays were performed to measure the expressions of thrombospondin-1 (THBS1), TGF-β1, PI3K, AKT and phosphorylated AKT (p-AKT) in the entrectinib-treated nerve cells. Additionally, we Preliminary observed and validated whether THBS1 overexpression could rescue nerve cell damage and the abnormalities in PI3K-AKT and TGF-β signaling pathways.

**Results:**

Entrectinib significantly inhibited the nerve cells proliferation and colony formation, and induced nerve cells apoptosis. Transcriptome sequencing analysis and qRT-PCR revealed that THBS1 was downregulated within entrectinib treatment. KEGG and GSEA analysis also suggested that entrectinib directly caused the abnormalities in proliferation-related signaling pathway like PI3K-AKT pathway, and apoptosis-related signaling pathway including TGF-β pathway. We further demonstrated that THBS1, TGF-β1, PI3K, AKT and p-AKT were downregulated by entrectinib. Meanwhile, pretreatment with THBS1 overexpression plasmids significantly rescued nerve cells (PC12, HT22 and SK-N-SH) from cell death and the abnormalities in PI3K-AKT and TGF-β signaling pathways.

**Conclusion:**

These results identified a critical role of entrectinib in promoting nerve cell damage by downregulating the expression of THBS1 while also inhibiting PI3K-AKT and TGF-β signaling pathways. Our findings will provide potential therapeutic targets for CNS-related toxicities.

## 1 Introduction

In recent years, cancer has become the leading cause of mortality worldwide. The increasing burden of cancer has become a challenging major public health problem (Tu et al., 2016; Jassim et al., 2023). Conventionally, there are three therapeutic modalities of cancer treatment such as surgical treatment, radiotherapy, and chemical drug therapy (Zheng et al., 2018; Yang et al., 2022). Nevertheless, the systemic toxicity associated with above therapies pose a significant challenge to patient tolerance and compliance (Mun et al., 2018). With the development and application of genetic testing in clinical treatment, molecular-targeting therapy has drawn widespread concern, bringing further options for clinical application (Bhamidipati and Subbiah, 2023; Li et al., 2023). Currently, targeted therapy has been an indispensable alternation to manage the disease for numerous cancers with oncogene addiction (Yuan et al., 2019; Benitez et al., 2021).

Entrectinib is an orally active small-molecule, which targets tyrosine kinase inhibitor of neurotrophic tyrosine receptor kinase (NTRK), ROS1 and anaplastic lymphoma kinase (ALK) genes. As the first-generation tyrosine receptor kinase inhibitor (TRKi), it is approved for the treatment of patients with NTRK fusion-positive solid tumors and adults with ROS1 fusion-positive non-small cell lung cancer (Frampton, 2021; Desai et al., 2022). Despite its impressive efficacy against cancer, the serious toxicities on the central nervous system (CNS) have also been popularly concerned by the public during the course of cancer therapy (Marcus et al., 2021). For example, the overall incidence of fatigue was 45%, taste disturbance was 42.3%, dysesthesia was 29.0%, cognitive impairment was 24.2% and peripheral sensory neuropathies was 18%. These symptoms not only significantly reduce the patient’s quality of life but also pose substantial challenges in the management of treatment, and even result in treatment interruptions (Trendowski et al., 2019; Doebele et al., 2020; Frampton, 2021; Martineau et al., 2022). Nowadays, these clinical CNS-related toxicities were only addressed through dose modification and interruptions, even withdrawal of entrectinib (Marcus et al., 2021). Unfortunately, there has been no effective protocols available for relieving or treating these symptoms until now. Thus, it is crucial to illuminate the molecular mechanism underlying CNS-related toxicities.

Research indicated that the tropomyosin receptor kinases (TRK) protein, a member of the tyrosine kinase family, plays a critical role in modulating neuronal activity and axonal growth in both the central and peripheral nervous systems (Aydin-Abidin and Abidin, 2019; Jiang et al., 2021). Acting as a tyrosine receptor kinase inhibitor (TRKi), entrectinib can inhibit TRK, thus leading to on-target neurotoxicity (Giustini et al., 2022). Some chemotherapy drugs can lead to chemotherapy-induced peripheral neuropathy which is involved in nerve damage and axon loss (Fumagalli et al., 2020; Chen et al., 2024). Several factors, such as mitochondrial dysfunction, oxidative stress, microcirculation disturbance and neuroinflammation have been proposed as determinants of neurotoxicity (Staff et al., 2017; Wang et al., 2023; Chen et al., 2024). Evidence indicated that isoflurane could induce hippocampal cells apoptosis by inhibiting PI3K-AKT expression (Wang et al., 2016). However, due to the complexity of CNS-related toxicities, the mechanisms underlying entrectinib-induced neurotoxicity are not fully understood.

In this study, we aim to evaluate whether entrectinib could induce nerve cell damage *in vitro* model. The potential mechanism responsible for entrectinib-induced nerve cell damage will be investigated as well. Furthermore, this finding will offer important insights and potential therapeutic targets for the entrectinib-induced nerve cell damage.

## 2 Materials and methods

### 2.1 Cell line and cell culture

The rat adrenal pheochromocytoma cells PC12 (FH0415), mouse hippocampal neuron cells HT22 (FH1027) and human neuroblastoma cells SK-N-SH (FH0164) were purchased from Shanghai Fuheng Biotechnology Co., Ltd. (Shanghai, China). PC12 cells were cultured in RPMI-1640 medium (KGM31800, KeyGEN, Nanjing, China) supplemented with 10% fetal bovine serum (FBS, A6901FBS, Invigentech, United States) and 1% penicillin and streptomycin (C0222, Beyotime, Shanghai, China). HT22 and SK-N-SH cells were cultured in DMEM medium (KGM12800, KeyGEN, Nanjing, China) with 10% FBS and 1% penicillin and streptomycin. All cells were incubated in a 37°C with 5% CO_2_ atmosphere.

### 2.2 Reagent and antibody

Entrectinib (HY-12678) was obtained from MedChemExpress (MCE, Shanghai, China) with the purity of 99.87%. THBS1 overexpression plasmids were synthesized by Corues Biotchnology (Nanjing, China). The relevant materials used in this study were provided as following: EnoGeneCell Counting Kit-8 (CCK-8, E1CK-000208, EnoGene, Nanjing, China), BeyoClick™ EdU-555 cell proliferation detection kit (C0075S, Beyotime, Shanghai, China), Annexin V-FITC/PI apoptosis detection kit (KGA107, KeyGEN, Nanjing, China), 4% paraformaldehyde (PFA, BL539A, Biosharp, Hefei, China), crystal violet (C0121, Beyotime, Shanghai, China), Trizol (R0016, Beyotime, Shanghai, China), Freezol reagent (R711-01, Vazyme, Nanjing, China), Evo M-MLV RT mix kit (AG11728, Accurate, Changsha, China), AceQ Universal SYBR qPCR master mix (Q511-02, Vazyme, Nanjing, China), BCA protein assay kit (P0010, Beyotime, Shanghai, China), RIPA (P00138, Beyotime, Shanghai, China), ECL detection reagent (180-501, Tanon, Shanghai, China), lipofectamine 3000 transfection reagent (L3000015, Invitrogen, United States).

The antibodies used in Western blotting assay were obtained as following: THBS1 (1:1,000, 18304-1-AP, Proteintech, Wuhan, China), PI3K (1:600, 20584-1-AP, Proteintech, Wuhan, China), AKT (1:5,000, 60203-2-AP, Proteintech, Wuhan, China), p-AKT (1:2,000, 4060T, Cell signaling technology, United States), TGF-β (1:3,000, 21898-1-AP, Proteintech, Wuhan, China), GAPDH (1:60,000, 60004-1-Ig, Proteintech, Wuhan, China), HRP-conjugated affinipure goat anti-rabbit IgG (1:5,000, SA00001-2, Proteintech, Wuhan, China) and HRP-conjugated affinipure goat anti-mouse IgG (1:4,000, SA00001-1, Proteintech, Wuhan, China).

### 2.3 Cells model establishment and treatment

To establish entrectinib activated cells, nerve cells (PC12, HT22 and SK-N-SH) were treated with entrectinib for 48 h at the concentrations of 0, 0.5, 1, 2, 5, 10, and 20 μmol/L, respectively. To explore whether entrectinib influences the cellular proliferation ability and apoptosis, we employed entrectinib (2.3, 4.2 and 4.3 μmol/L, respectively) to the PC12, HT22 and SK-N-SH cells for 48 h. To observe the effect of THBS1 on entrectinib activated cells, nerve cells were divided into five groups: the normal control (NC), the overexpression (OE) group transfected with THBS1 overexpression plasmids, the entrectinib group exposed to entrectinib (2.3, 4.2 and 4.3 μmol/L, respectively), the NC + entrectinib group and the THBS1 overexpression group cultured in medium containing entrectinib (2.3, 4.2 and 4.3 μmol/L, respectively) for 48 h.

### 2.4 CCK-8 assay

PC12, HT22 and SK-N-SH cells were plated in 96-well plates at a density of 7 × 10^3^ cells per well and incubated for 24 h. Subsequently, PC12, HT22 and SK-N-SH cells were treated with entrectinib and THBS1 overexpression plasmids for 48 h described above. After incubation, 10 μL CCK-8 solution was added into each well and incubated at 37°C for 2 h in the dark. Then, a microplate reader (Molecular Devices, Shanghai, China) was used to measure the absorbance at a wavelength of 450 nm.

### 2.5 EdU incorporation assay

PC12, HT22 and SK-N-SH cells were seeded into 24-well plates at a density of 3 × 10^4^ cells per well and treated with entrectinib for 48 h. BeyoClick™ EdU-555 cell proliferation detection kit was used for the subsequent experiments. In brief, 20 μmol/L EdU was added into per well for 4 h. Hoechst 33342 was used to stain the nuclei for 10 min in the dark. Next, images were randomly captured by a fluorescence inverted microscope (Zeiss, Germany). Finally, ImageJ was utilized to count the total cell and proliferating cell numbers.

### 2.6 Colony formation assay

In six-well plates, PC12, HT22, SK-N-SH cells were cultured at a density of about 7 × 10^2^ cells per well and then treated with entrectinib. The cultures were maintained for 14 days or until the number of single-cell clones exceeded 50. After rinsing with phosphate buffered saline (PBS), cells were fixed with 4% paraformaldehyde solution for 30 min. Next, 0.5% crystal violet solution was stained for 15 min. Finally, cells were washed several times with PBS and captured by a camera.

### 2.7 Flow cytometry analysis

PC12, HT22 and SK-N-SH cells were seeded into 6-well plates and treated with entrectinib and THBS1 overexpression plasmids for 48 h described above. Then, the treated cells were collected and stained with Annexin V-FITC and PI staining solution for 10 min. Following the recommendations of the manufacturer, the cells were analyzed by flow cytometry (BD Biosciences, United States) within 1 h. Data were analyzed by Flowjo software (v.10.8.1).

### 2.8 Gene differential expression analysis

PC12, HT22 and SK-N-SH cells were treated with entrectinib, respectively. After 48 h incubation, the total RNA was extracted by Trizol reagent and sequenced in Beijing Biomarker Technology Co., LTD. (Beijing, China). Briefly, according to the manufacturer’s instructions, the sequencing library was generated by Hieff NGS ultima dual-mode mRNA library prep kit (Yeasen, Shanghai, China). The libraries were quantified preliminarily with Qubit 3.0 fluorescence quantitative analyzer and sequenced on an Illumina novaseq platform to generate 150 bp paired-end reads. Clean data were obtained by removing reads containing adapter, ploy-N and low-quality from raw data. The analysis of differentially expressed genes (DEGs) between PC12 and HT22 cells was performed by DESeq2 software (Love et al., 2014), while SK-N-SH cells were analyzed by edgeR software (Robinson et al., 2010). Genes with a statistical threshold of false discovery rate (FDR) < 0.05 and |fold change| > 2 were considered to be significantly differentially expressed. Volcano plots, a visualized plots to illustrate DEGs, were generated by bioinformatics software available at https://www.bioinformatics.com.cn/.

### 2.9 Gene ontology (GO) and kyoto encyclopedia of genes and genomes (KEGG) analysis

Functional enrichment analyses of GO (Ashburner et al., 2000) and KEGG (Kanehisa et al., 2014) in DEGs from PC12 and HT22 cells were performed by the R package ClusterProfiler (v.4.6.2) (Yu et al., 2012). The R package ggplot2 (v.3.4.1) was utilized to visualize and screen biological functions and pathways associated with these DEGs. Enriched GO terms and KEGG pathways were presented to illustrate the analytical outcomes.

### 2.10 Gene set enrichment analysis (GSEA) analysis

GSEA software (v.4.3.2) (Subramanian et al., 2005) was performed to investigate functional enrichment pathways and landmark gene sets in gene expression datasets. Signature gene sets were extracted from the Molecular Signatures Database (MSigDB, available at https://www.gsea-msigdb.org). Enrichment results were considered significant if the normalized enrichment score (NES) > 1 and the nominal *P <* 0.05 in the entrectinib treatment.

### 2.11 RNA extraction and quantitative real time polymerase chain reaction (qRT-PCR)

According to the manufacturer’s recommendations, total cellular RNA was extracted by Freezol reagent and reverse-transcribed into cDNA by the Evo M-MLV RT mix kit. The AceQ universal SYBR qPCR master mix kit was used for quantitative real-time PCR. The β-actin was served as the internal control. The expression of the target gene was calculated by the 2^−ΔΔCT^ method. Primers used in this study were synthesized by Invitrogen Biotechnology Co., Ltd. (Invitrogen, United States), and their sequences were provided in Supplementary Table S1.

### 2.12 Western blotting assay

Total protein from cells was extracted through RIPA lysate with a mixture of phosphatase inhibitor and protease inhibitor. The concentration of extracted proteins was quantified through BCA protein assay kit. The protein samples were separated through 10% sodium dodecyl sulfate polyacrylamide gel electrophoresis (SDS-PAGE) and transferred to PVDF membranes. After blocking with 5% skim milk for 2 h, the PVDF membranes were incubated with the primary antibody overnight at 4°C. Subsequently, membranes were incubated with the corresponding secondary antibody and visualized by an enhanced chemiluminescence (ECL) reagent. Protein band intensities were analyzed by ImageJ software.

### 2.13 Statistical analysis

All experiments were conducted at least in triplicate to ensure reliability. Statistical analysis and graphical representation were carried out by GraphPad Prism (v.9.0). Differences in quantitative variables between two groups were assessed by the t-test, while differences among three or more groups were evaluated by one-way analysis of variance (ANOVA). *P <* 0.05 was considered as statistical significance.

## 3 Results

### 3.1 Entrectinib significantly inhibited the viability of nerve cells (PC12, HT22 and SK-N-SH)

**FIGURE 1:**
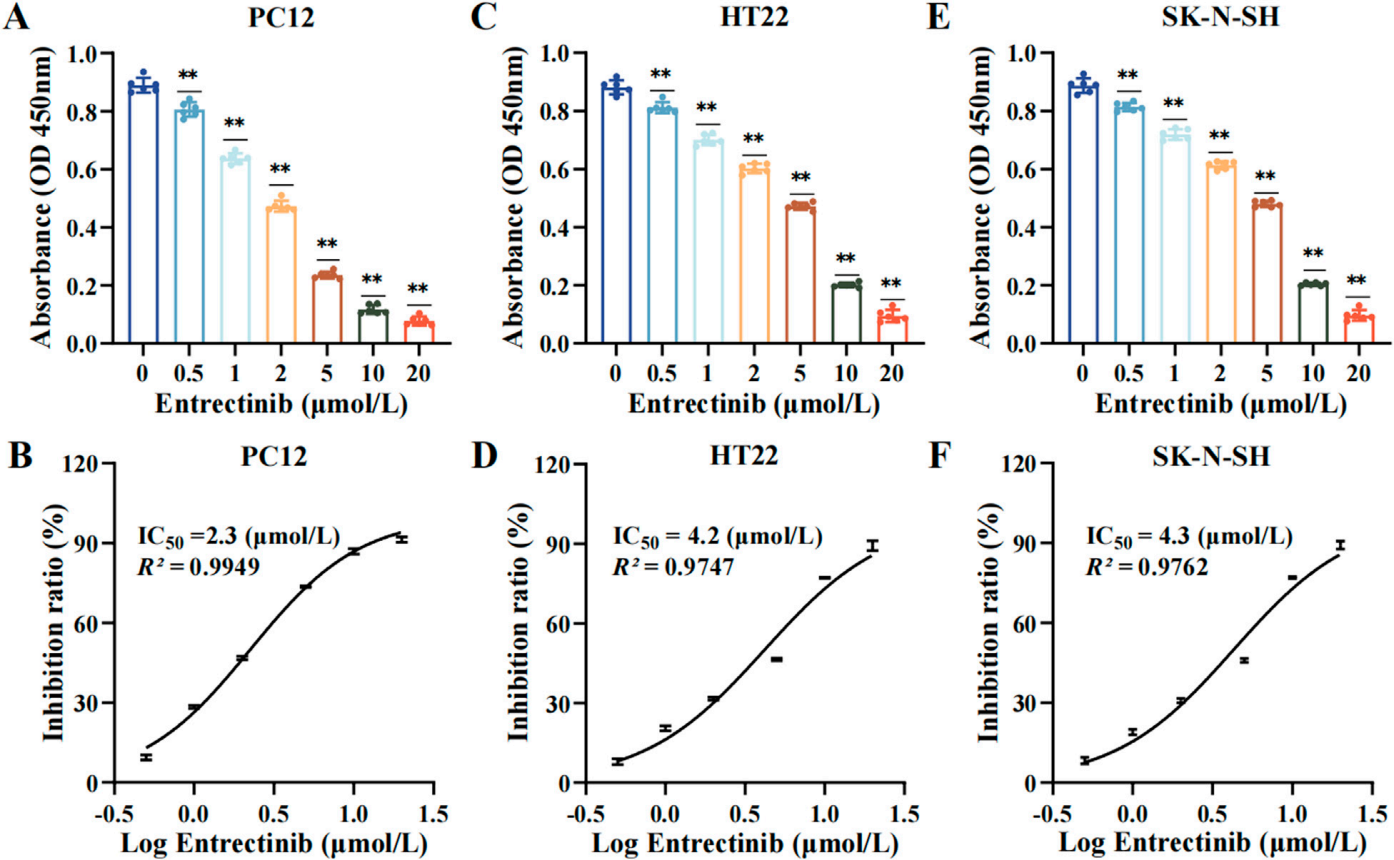
Entrectinib significantly inhibited nerve cells viability. **(A, C, E)** The absorbance of PC12, HT22 and SK-N-SH cells was subjected to the CCK-8 assay after treatment with entrectinib (0, 0.5, 1, 2, 5, 10, and 20 μmol/L). Data are shown as mean ± SD, *n =* 6 (^**^ *P <* 0.01). **(B, D, F)** The IC_50_ value of PC12, HT22 and SK-N-SH cells was calculated by GraphPad Prism (v.9.0). Data are shown as mean ± SD, *n =* 6.

Initially, the nerve cells (PC12, HT22 and SK-N-SH) were subjected to various concentrations of entrectinib (0, 0.5, 1, 2, 5, 10, and 20 μmol/L) for 48 h, and the impact of entrectinib on nerve cells viability was assessed by the CCK-8 assay. The results indicated that entrectinib significantly inhibited nerve cells viability compared with the control group, displaying a clear dose-dependent reduction trend (Figures 1A, C, E). Subsequently, the IC_50_ of entrectinib in PC12, HT22 and SK-N-SH cells was calculated as 2.3 μmol/L, 4.2 μmol/L, and 4.3 μmol/L, respectively (Figures 1B, D, F). Thus, the IC_50_ entrectinib incubation was selected for subsequent experiments. Collectively, these results preliminarily indicated that entrectinib showed an inhibitory effect on the nerve cells viability.

### 3.2 Entrectinib significantly inhibited nerve cells proliferation

**FIGURE 2:**
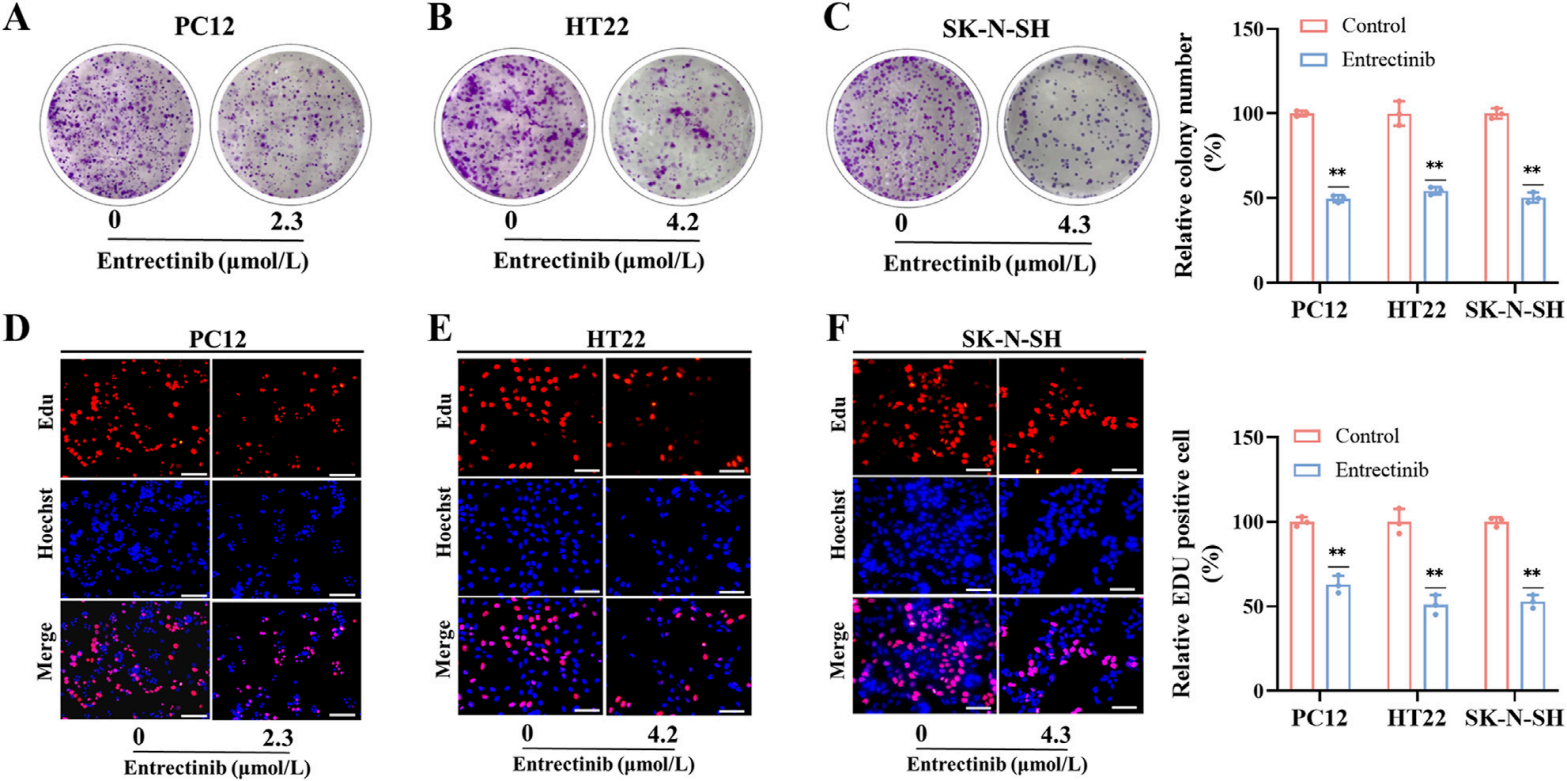
Entrectinib inhibited nerve cells proliferation. **(A–C)** PC12, HT22 and SK-N-SH cells treated with entrectinib (2.3, 4.2, and 4.3 μmol/L, respectively) were seeded into 6-plate wells, and the number of colonies was counted on the 14 days. The results are also shown in the bar chart. Data are shown as mean ± SD, *n =* 3 (^**^ *P <* 0.01). **(D–F)** EdU incorporation assay was performed to determine the nerve cells proliferation ability of PC12, HT22 and SK-N-SH cells treated with entrectinib (2.3, 4.2, and 4.3 μmol/L, respectively), and the results are also shown in the bar chart. Scale bar = 40 μm. Data are shown as mean ± SD, *n =* 3 (^**^ *P <* 0.01).

To further investigate entrectinib’s impact on nerve cell proliferation, we performed colony formation and EdU incorporation assays. As shown in Figures 2A–C, the relative colony number of nerve cells (PC12, HT22, SK-N-SH) was significantly decreased to 49.65%, 54.34%, and 50.21% after entrectinib treatment. In addition, EdU incorporation assay demonstrated that the red fluorescence had a notable reduction in the treatment of entrectinib, indicating that entrectinib could inhibit the replicative capacity of nerve cells (Figures 2D–F). These findings confirmed that entrectinib significantly inhibited the proliferation ability of nerve cells.

### 3.3 Entrectinib could induce nerve cells apoptosis

**FIGURE 3:**
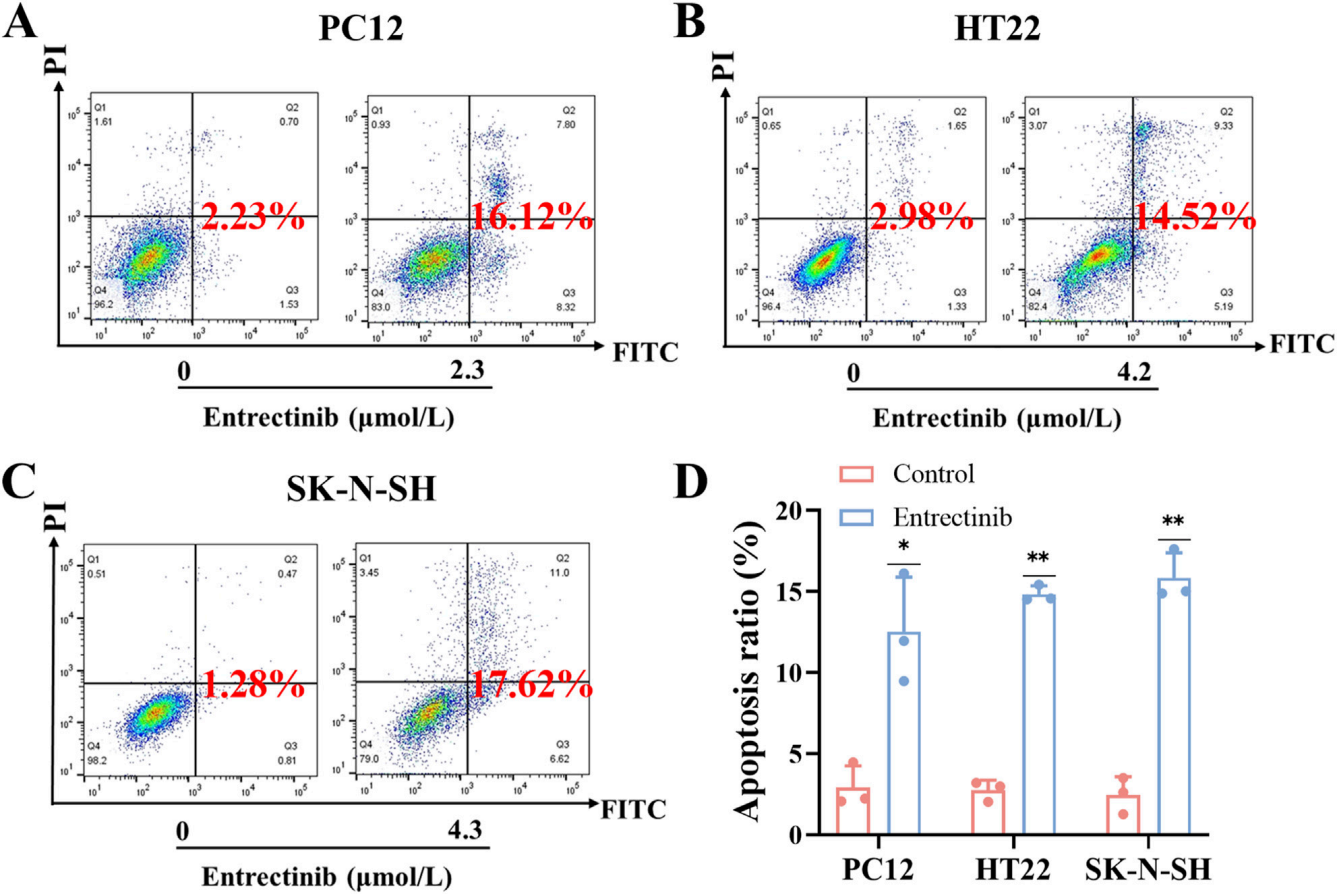
Entrectinib induced nerve cells apoptosis. **(A–C)** The effect of entrectinib on nerve cells (PC12, HT22 and SK-N-SH) apoptosis was measured by flow cytometry analysis. **(D)** Quantitative mean apoptosis ratio of **(A–C)**. Data are shown as mean ± SD, *n =* 3 (^**^ *P <* 0.01).

Moreover, Annexin V-FITC/PI apoptosis detection kit was employed to further explore the impact of entrectinib on nerve cells apoptosis. PC12, HT22 and SK-N-SH cells were treated with entrectinib of 2.3 μmol/L, 4.2 μmol/L and 4.3 μmol/L, respectively, and the apoptosis rate was significantly increased to 12.52%, 14.83% and 15.84% (Figures 3A–D). Taken together, these results indicated that entrectinib could significantly inhibit the viability and induce apoptosis within the nerve cells.

### 3.4 Identification of DEGs in PC12, HT22 and SK-N-SH cells

**FIGURE 4:**
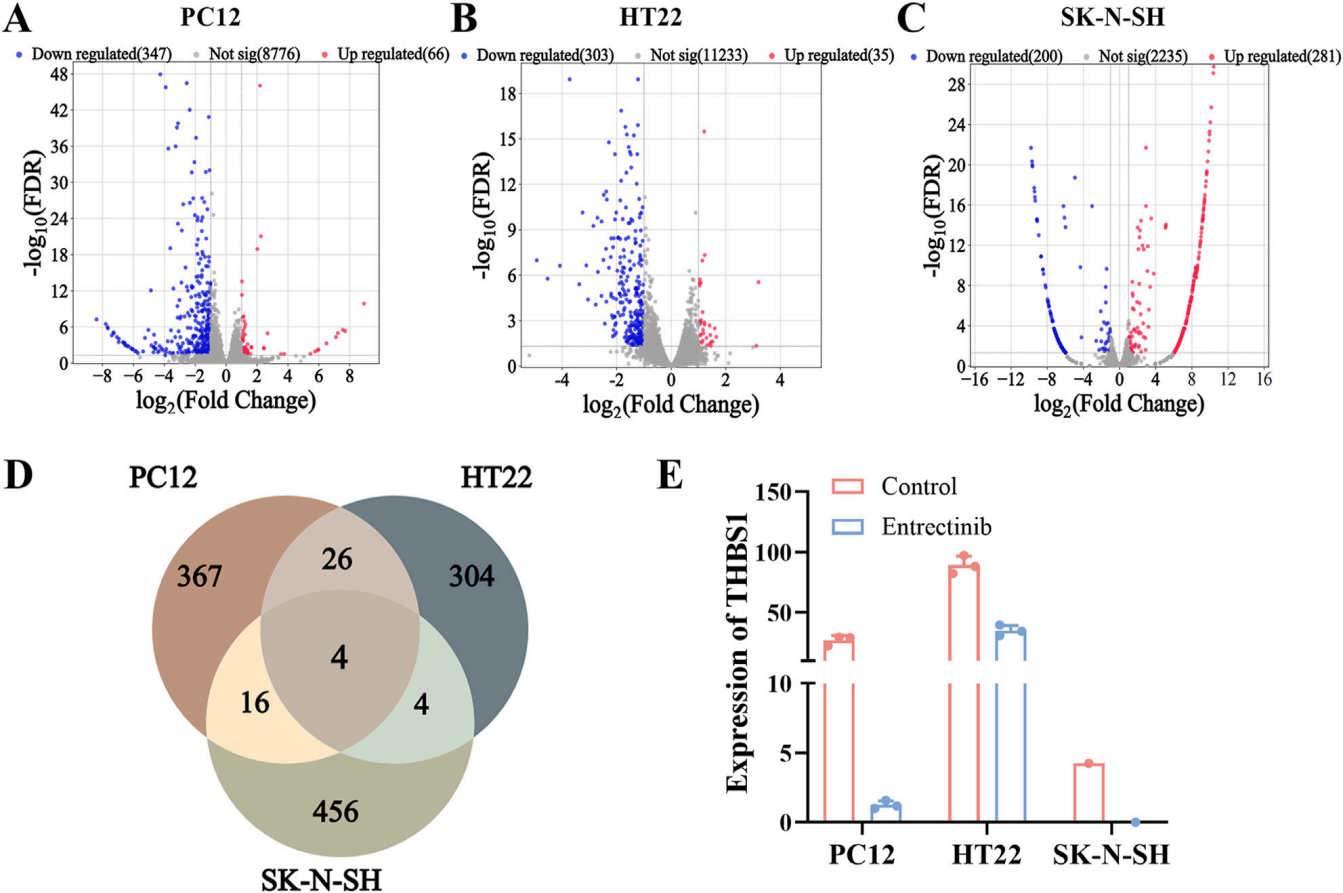
Transcriptome sequencing analyses identified THBS1 target involved in nerve cell damage. **(A–C)** The DEGs of PC12, HT22 and SK-N-SH cells were presented in the form of volcano plot, where red represents upregulated genes, blue represents downregulated genes, and gray represents genes with insignificant differences (Set threshold FDR < 0.05 and |fold change| > 2). **(D)** Venn diagram was performed to illustrate overlap between DEGs by PC12, HT22 and SK-N-SH cells. **(E)** The expression of THBS1 was identified by transcriptome sequencing analyses.

To further explore the impact of entrectinib on nerve cells transcription levels, we employed high-throughput sequencing technology on the entrectinib-treated nerve cells (PC12, HT22 and SK-N-SH) samples. Differential expression analysis revealed that 413, 338, and 481 DEGs were in PC12, HT22 and SK-N-SH cells, respectively (Figures 4A–C; Supplementary Table S2). We then determined the intersection of DEGs present in the nerve cells, where 4 DEGs (FTL1, THBS1, FTL1-PS1, COL3A1) were intersected (Figure 4D). Notably, thrombospondin-1 (THBS1) was significantly downregulated in entrectinib treatment, implying that it might be involved in nerve cell damage (Figure 4E). These findings confirmed that entrectinib could induce extensive transcriptomic changes, and THBS1 was the most significant.

### 3.5 Exploration the functions and pathways of DEGs

**FIGURE 5:**
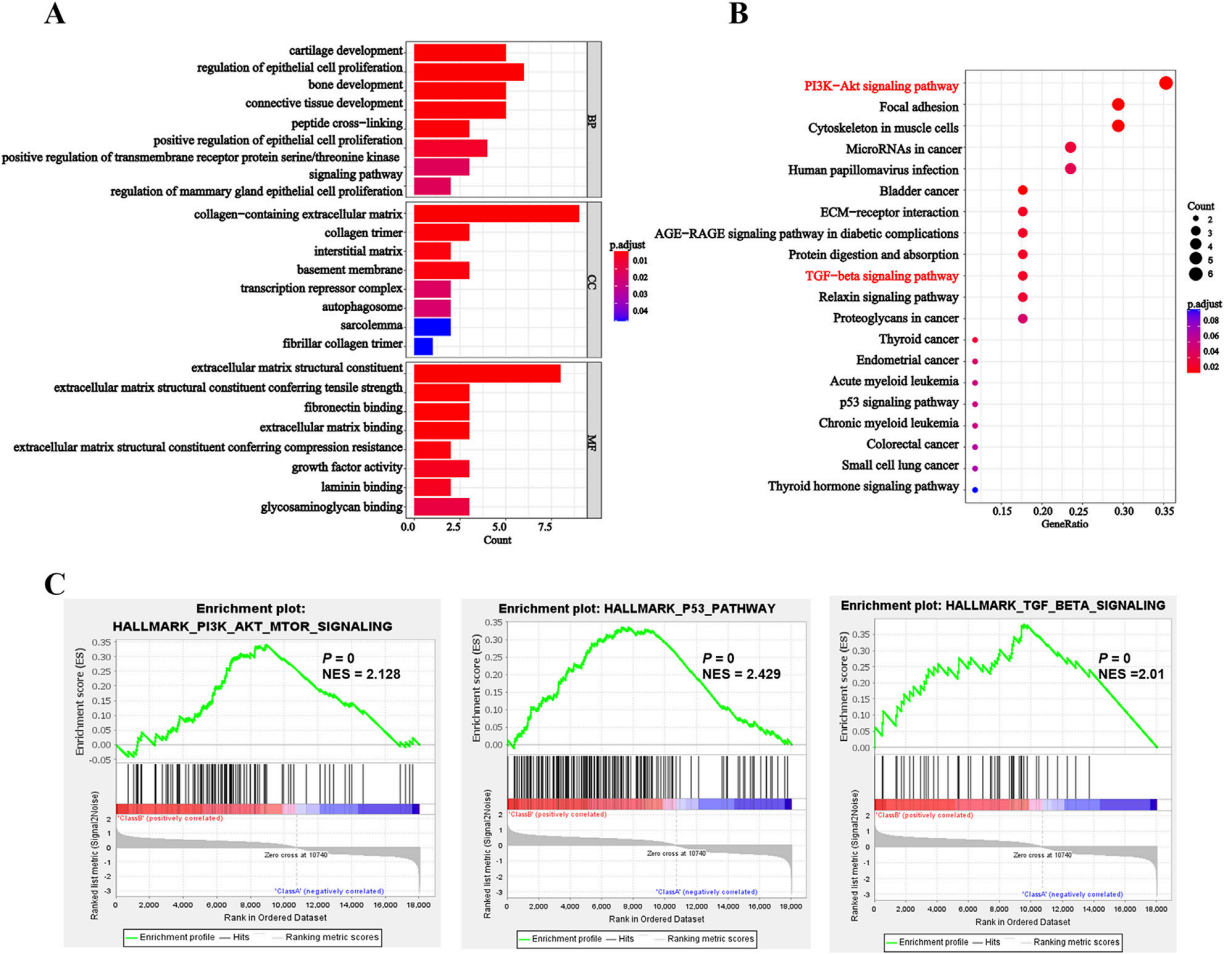
Entrectinib induced the nerve cell damage by affecting the PI3K-AKT-mTOR signaling pathway, p53 signaling pathway and TGF-β signaling pathway. **(A, B)** The R package ClusterProfiler (v.4.6.2) was used for clustering analysis of potential targets and pathways of entrectinib-induced nerve cell damage. Histogram and bubble plots showed the results of GO and KEGG enrichment analysis. **(C)** GSEA of hallmark gene sets between control groups and entrectinib-treated groups.

Next, we conducted GO and KEGG pathway enrichment analyses to uncover the biological functions and pathways associated with DEGs. GO results revealed that DEGs were involved in biological processes including peptide cross-linking and epithelial cell proliferation regulation. Meanwhile, it also showed the relationship with cellular components and molecular functions, such as extracellular matrix, transcription repressor complex, growth factor activity and extracellular matrix binding (Figure 5A; Supplementary Table S3). Futhermore, KEGG analysis implicated that DEGs were enriched in pathways such as PI3K-AKT signaling pathway, TGF-β signaling pathway and p53 signaling pathway, et al. (Figure 5B; Supplementary Table S4). Similarly, GSEA was also performed across hallmark gene sets to identify potential signatures of response. The results showed that PI3K-AKT-mTOR signaling pathway, TGF-β signaling pathway and p53 signaling pathway (Figure 5C; Supplementary Table S5) were enriched in entrectinib treatment. The above results suggested that the entrectinib-induced nerve cell damage may be related to the PI3K-AKT-mTOR signaling pathway, p53 signaling pathway and TGF-β signaling pathway.

### 3.6 Entrectinib could downregulate THBS1 expression while also inhibiting PI3K-AKT and TGF-β signaling pathways

**FIGURE 6:**
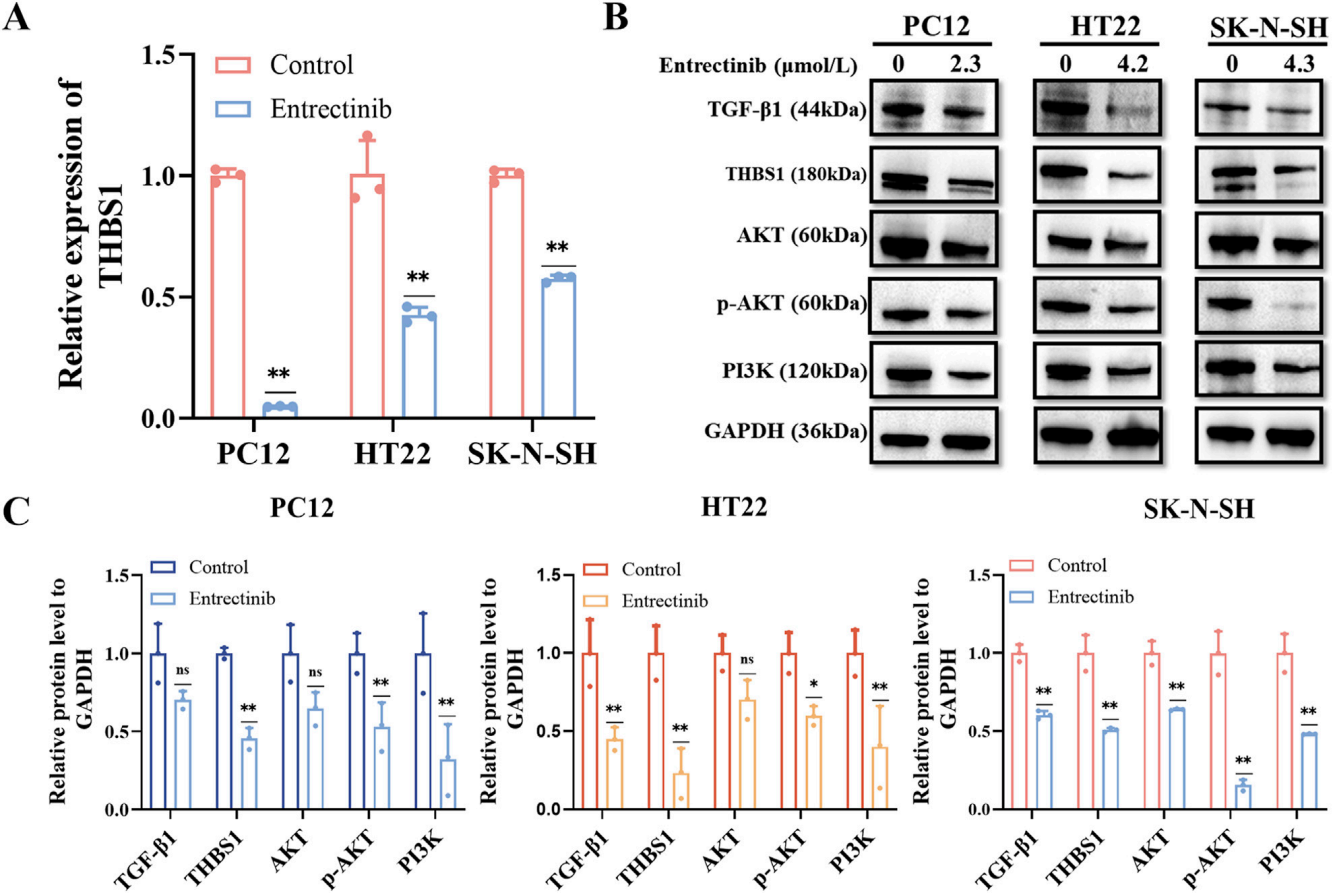
Entrectinib downregulated THBS1 expression while also inhibiting PI3K-AKT and TGF-β signaling pathways. **(A)** The expression of THBS1 in PC12, HT22 and SK-N-SH cells treated with entrectinib was detected by qRT-PCR. Data are shown as mean ± SD, *n* = 3 (^**^ *P <* 0.01). **(B)** The expressions of PI3K, AKT, p-AKT, TGF-β and THBS1 proteins in PC12, HT22, SK-N-SH cells treated with entrectinib were detected by Western blotting assay. **(C)** The quantitative analyses of TGF-β1, THBS1, AKT, p-AKT and PI3K proteins in **(B)** are shown. Data are shown as mean ± SD, *n* = 3 (^*^ *P <* 0.05, ^**^ *P <* 0.01).

To validate the transcriptome sequencing analysis results, we further performed qRT-PCR and Western blotting assays. Compared with the control group, the expression of THBS1 was significantly decreased after entrectinib treatment within the nerve cells (Figures 6A, B), which was in parallel with sequencing results. Additionally, the levels of relative proteins involved in PI3K-AKT and TGF-β signaling pathways were examined as well. Obviously, entrectinib could downregulate the expressions of PI3K, AKT, phosphorylated AKT (p-AKT) and TGF-β1 proteins within the nerve cells (PC12, HT22 and SK-N-SH) (Figures 6B–C). Therefore, these results demonstrated that entrectinib could downregulate THBS1 expression while also inhibiting PI3K-AKT and TGF-β signaling pathways.

### 3.7 THBS1 overexpression could rescue nerve cell damage induced by entrectinib and activate PI3K-AKT and TGF-β signaling pathways

**FIGURE 7:**
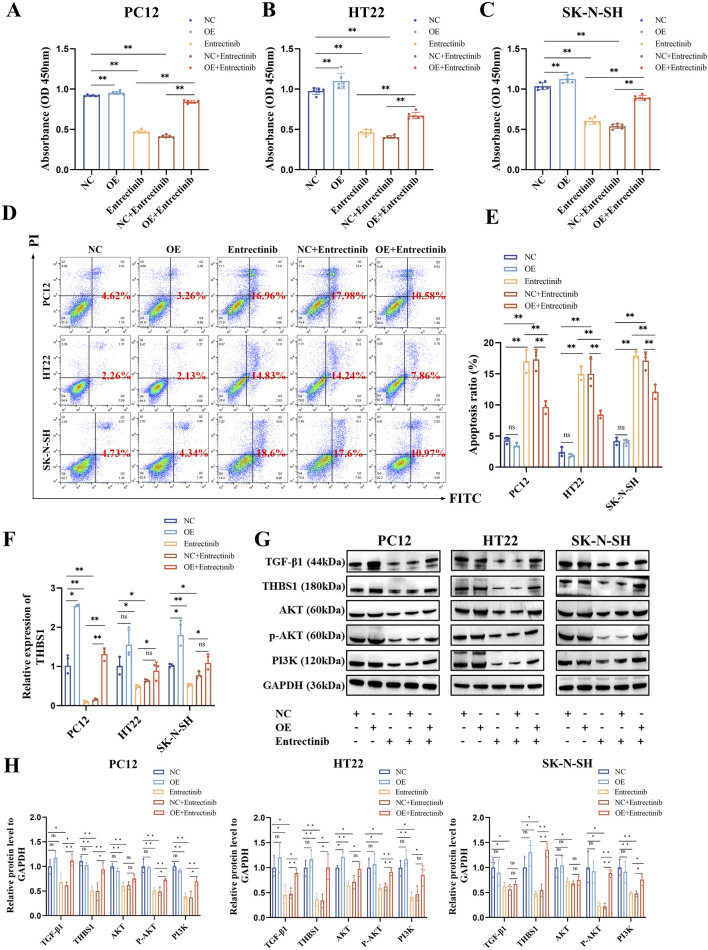
THBS1 overexpression rescue nerve cell damage and the abnormalities in the PI3K-AKT and TGF-β signaling pathways induced by entrectinib. **(A–C)** Overexpression of THBS1 in entrectinib-treated cells rescued cell viability. Data are shown as mean ± SD, *n =* 6 (^**^ *P <* 0.01). **(D, E)** Overexpression of THBS1 in entrectinib-treated cells rescued cell apoptosis. Data are shown as mean ± SD, *n =* 3 (^**^ *P <* 0.01). **(F)** qRT-PCR measured the efficiency of THBS1 overexpression. Data are shown as mean ± SD, *n* = 3 (^*^ *P <* 0.05, ^**^ *P <* 0.01). **(G)** Western blotting assay examined the levels of PI3K, AKT, p-AKT, TGF-β and THBS1 proteins in PC12, HT22, SK-N-SH cells after treatement with entrectinib or THBS1 overexpression plasmids or the combination for 48 h. **(H)** Representative quantification were shown. Data are shown as mean ± SD, *n* = 3 (^*^ *P <* 0.05, ^**^ *P <* 0.01).

To preliminary observe and validate whether THBS1 overexpression could rescue nerve cell damage and the abnormalities in PI3K-AKT and TGF-β signaling pathways, nerve cells were transfected with THBS1 overexpression plasmids. As shown in Figures 7A–E, THBS1 overexpression can rescue nerve cell damage induced by entrectinib. Additionally, the expressions of PI3K, AKT, phosphorylated AKT (p-AKT) and TGF-β1 proteins were also upregulated by THBS1 overexpression in the entrectinib-treated cells (Figures 7F–H). These above results suggested that THBS1 plays an important functional role in rescuing nerve cell damage and the abnormalities in the PI3K-AKT and TGF-β signaling pathways induced by entrectinib.

## 4 Discussions

Entrectinib, a novel multi-target TRKi, has demonstrated considerable therapeutic efficacy in tumors harboring NTRK, ROS1 or ALK gene fusions (Desai et al., 2022). It can penetrate the blood-brain barrier and exhibit CNS activity, underscoring its potential in treating brain tumors (Liu et al., 2018; Desai et al., 2022; Jiang et al., 2022). However, bulk evidences suggest that entrectinib can cause serious neurotoxicity following prolonged application, such as sensory neuropathy and peripheral neuropathy (Delgado et al., 2021; Giustini et al., 2022). However, the underlying mechanism of entrectinib-induced neurotoxicity remains elusive.

In this study, we investigated the effects of entrectinib on PC12, HT22 and SK-N-SH cells *in vitro*, focusing on its impact on the proliferation and apoptosis of nerve cells. Initially, a CCK-8 assay was carried out to determine cellular viability, we demonstrated that entrectinib inhibited the cell viability in a dose-dependent trend. Furthermore, EdU incorporation and colony formation assays were used to analyze the nerve cells proliferation. We also observed a significant inhibition of cell proliferation and clonogenic potential after entrectinib administration, confirming its anti-proliferative effects *in vitro*. Flow cytometry analysis further demonstrated that entrectinib effectively increased the apoptosis ratio of nerve cells. Taken together, these results indicated that entrectinib could significantly inhibit proliferation ability and induce apoptosis within the nerve cells.

Subsequently, transcriptome sequencing was performed to explore the gene expression changes of entrectinib-treated nerve cells. The transcriptome sequencing analysis identified THBS1 was intersected by PC12, HT22 and SK-N-SH cells, and it showed a downregulated trend. THBS1 belongs to adhesion glycoprotein, which plays a pivotal role in mediating cell-cell and cell-matrix interactions (Huang et al., 2017; Yao et al., 2023). THBS1, first discovered in platelets, but now many studies indicate that it plays a crucial role in the development of diseases (Firlej et al., 2011). Moreover, THBS1 is also involved in the physiological and pathological processes of the nervous system and indispensable for axon regeneration (Bray et al., 2019; Yao et al., 2023). Therefore, we further examined the expression of THBS1 within entrectinib treatment. The results revealed that the expression of THBS1 was significantly decreased by entrectinib, which was in parallel with sequencing results. In addition, THBS1 overexpression rescued entrectinib-induced nerve cell damage. Collectively, these results indicated that entrectinib-induced nerve cell damage may be related to the downregulation of THBS1.

KEGG and GSEA analysis results revealed that the PI3K-AKT and TGF-β signaling pathways were significantly enriched in entrectinib treatment. The PI3K-AKT signaling pathway plays an important role in intracellular signal transduction and regulates diverse cellular processes including cell cycle, adhesion, migration, inflammation, metabolism and survival (Jafari et al., 2019; Xiao et al., 2022). In addition, the PI3K-AKT signaling pathway also exerts profound influence on nervous system physiology, governing processes such as myelin formation (Gaesser and Fyffe-Maricich, 2016), axon regeneration (Huang et al., 2019), nerve cell regeneration (Luo et al., 2019) and apoptosis (Wang et al., 2022; Kilic et al., 2017; Lu et al., 2022). It was reported that PI3K-AKT signaling pathway could regulate neurotoxicity and mediate the survival of neurons (Goyal et al., 2023). Receptor tyrosine kinase (RTK), serving as principal upstream regulator of the PI3K-AKT pathway, modulated AKT activity through activating PI3K (Haddadi et al., 2018; Rai et al., 2019). It is worth noting that PI3K, as a crucial anti-apoptotic regulator, triggers the activation of its downstream target AKT upon its activation. Phosphorylation of transmembrane receptors such as RTK can lead to the activation of PI3K and p-AKT, thus activating neuroprotective effects (Griffin et al., 2005; Wang et al., 2022). Therefore, Western blotting assay was performed to examine the protein levels of PI3K, AKT and p-AKT within entrectinib treatment, we found that entrectinib could downregulate the levels of these proteins. Moreover, THBS1 overexpression can rescue the levels of relative proteins involved in PI3K-AKT signaling pathways in the entrectinib-treated nerve cells (PC12, HT22 and SK-N-SH). Thus, the reduction of the PI3K-AKT signaling pathway caused by THBS1 inhibition may be related to the entrectinib-induced nerve cell damage.

As we all known, THBS1 is a potential upstream target with TGF-β signaling pathway (Sun et al., 2022). TGF-β is a multifunctional peptide that governs the diverse cellular processes like cell proliferation, differentiation, death and migration (Jakowlew, 2006; Hata and Chen, 2016; Deng et al., 2024; Giarratana et al., 2024). TGF-β signaling pathway is also involved in neurotrophic signaling transmission and is closely related to the normal development and function of nerves (Meyers and Kessler, 2017; Ding et al., 2024). Brionne et al. (2003) research showed that the downregulation of TGF-β1 in primary neurons resulted in a strong reduction of survival. In addition, TGF-β1 heterozygous knockout mice displayed heightened sensitivity to toxic insults. THBS1 serves as a key activator of TGF-β, significantly activates TGF-β1 factor and TGF-β signaling pathway (Atanasova et al., 2019; Bedolla et al., 2024). Additionally, previous publications have reported that TGF-β signaling pathway induced renal injury may be related to the regulation of THBS1 (Sun et al., 2022). Taken together, we conducted qPCR and Western blotting assays to examine the expressions of THBS1 and TGF-β1 within entrectinib treatment. We found that entrectinib significantly downregulated the expressions of THBS1 and TGF-β1. Additionally, THBS1 overexpression can rescue TGF-β1 expression in the entrectinib-treated nerve cells, implicating that the reduction of the TGF-β signaling pathway caused by THBS1 inhibition may be a potential mechanism for the entrectinib-induced nerve cell damage.

## 5 Conclusion

**FIGURE 8:**
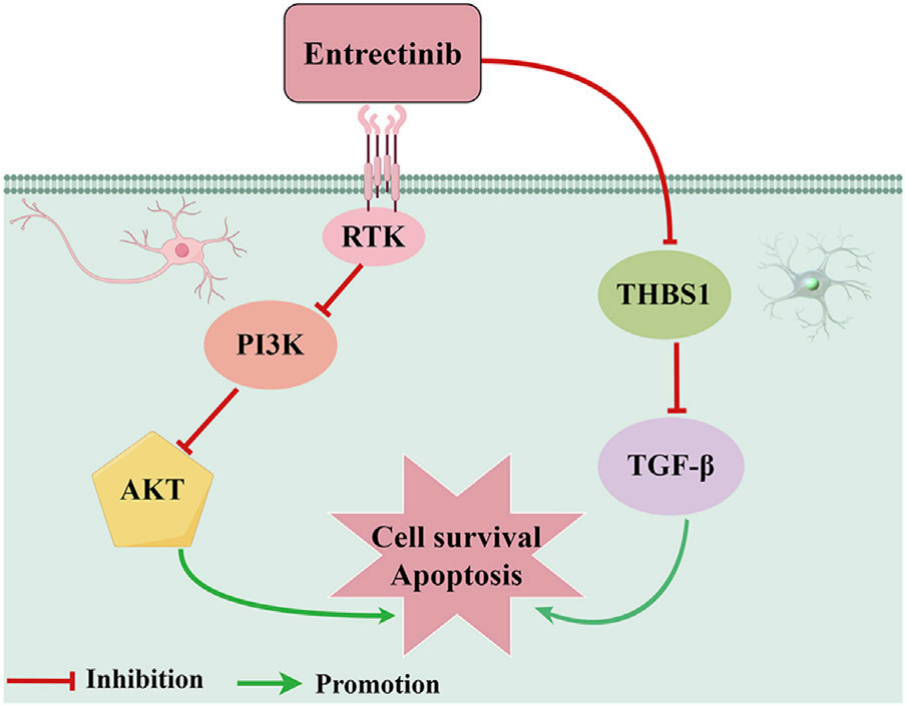
Proposed action pathway of entrectinib in inducing nerve cell damage. Entrectinib induced nerve cell damage by downregulating THBS1 expression while also inhibiting PI3K-AKT and TGF-β signaling pathways. The figure was drawn by Figdraw.

In conclusion, our findings proposed a putative mechanism whereby entrectinib-induced nerve cell damage may downregulate THBS1 expression while also inhibiting PI3K-AKT and TGF-β signaling pathways (Figure 8). Although our current study offers valuable insights, there still exists some limitations. Due to the limited availability of appropriate animal models, we were unable to investigate the effects of entrectinib on the proteins and gene expression levels within the nerve cells *in vivo*. Thus, future studies should aim to comprehensively illustrate the intricate mechanism based on *in vivo* experiments. In a word, our study contributed important insights into the molecular mechanism underlying entrectinib’s neurotoxic effects and proposes potential therapeutic targets.

## Data Availability

The raw data presented in the study are deposited in the Sequence Read Archive (SRA) repository, accession number is PRJNA1219491.

## Supplementary material

SUPPLEMENTARY TABLE S1 The qPCR primers used in this study.

SUPPLEMENTARY TABLE S2 The differentially expressed genes between control groups and entrectinib-treated groups in this study.

SUPPLEMENTARY TABLE S3 The results of GO analysis in this study.

SUPPLEMENTARY TABLE S4 The results of KEGG analysis in this study.

SUPPLEMENTARY TABLE S5 The results of GSEA analysis in this study.

SUPPLEMENTARY TABLE S6 The raw data of transcriptome sequencing in this study.

## References

[B1] Ashburner M. Ball C. A. Blake J. A. Botstein D. Butler H. Cherry J. M. (2000). Gene ontology: tool for the unification of biology. The Gene Ontology Consortium. Nat. Genet. 25 (1), 25–29. 10.1038/75556 10802651 PMC3037419

[B2] Atanasova V. S. Russell R. J. Webster T. G. Cao Q. Agarwal P. Lim Y. Z. (2019). Thrombospondin-1 is a major activator of TGF-β signaling in recessive dystrophic epidermolysis bullosa fibroblasts. J. Invest. Dermatol. 139 (7), 1497–1505.e5. 10.1016/j.jid.2019.01.011 30684555

[B3] Aydin-Abidin S. Abidin I. (2019). 7,8-Dihydroxyflavone potentiates ongoing epileptiform activity in mice brain slices. Neurosci. Lett. 703, 25–31. 10.1016/j.neulet.2019.03.013 30880161

[B4] Bedolla A. Wegman E. Weed M. Stevens M. K. Ware K. Paranjpe A. (2024). Adult microglial TGFβ1 is required for microglia homeostasis via an autocrine mechanism to maintain cognitive function in mice. Nat. Commun. 15 (1), 5306. 10.1038/s41467-024-49596-0 38906887 PMC11192737

[B5] Benitez J. C. Geraud A. Texier M. Massard C. Paci A. Soria J. C. (2021). Late phase 1 studies: concepts and outcomes. Lancet Oncol. 22 (10), e446–e455. 10.1016/S1470-2045(21)00467-8 34592194

[B6] Bhamidipati D. Subbiah V. (2023). Impact of tissue-agnostic approvals for patients with gastrointestinal malignancies. Trends Cancer 9 (3), 237–249. 10.1016/j.trecan.2022.11.003 36494311 PMC9974757

[B7] Bray E. R. Yungher B. J. Levay K. Ribeiro M. Dvoryanchikov G. Ayupe A. C. (2019). Thrombospondin-1 mediates axon regeneration in retinal ganglion cells. Neuron 103 (4), 642–657.e7. 10.1016/j.neuron.2019.05.044 31255486 PMC6706310

[B8] Brionne T. C. Tesseur I. Masliah E. Wyss-Coray T. (2003). Loss of TGF-beta 1 leads to increased neuronal cell death and microgliosis in mouse brain. Neuron 40 (6), 1133–1145. 10.1016/s0896-6273(03)00766-9 14687548

[B9] Chen X. Gan Y. Au N. P. B. Ma C. H. E. (2024). Current understanding of the molecular mechanisms of chemotherapy-induced peripheral neuropathy. Front. Molec. Neurosci. 17, 1345811. 10.3389/fnmol.2024.1345811 38660386 PMC11039947

[B10] Delgado J. Pean E. Melchiorri D. Migali C. Josephson F. Enzmann H. (2021). The European Medicines Agency review of entrectinib for the treatment of adult or paediatric patients with solid tumours who have a neurotrophic tyrosine receptor kinase gene fusions and adult patients with non-small-cell lung cancer harbouring ROS1 rearrangements. ESMO Open 6 (2), 100087. 10.1016/j.esmoop.2021.100087 33735800 PMC7988279

[B11] Deng Z. Fan T. Xiao C. Tian H. Zheng Y. Li C. (2024). TGF-β signaling in health, disease, and therapeutics. Signal Transduct. Target. Ther. 9 (1), 61. 10.1038/s41392-024-01764-w 38514615 PMC10958066

[B12] Desai A. V. Robinson G. W. Gauvain K. Basu E. M. Macy M. E. Maese L. (2022). Entrectinib in children and young adults with solid or primary CNS tumors harboring NTRK, ROS1, or ALK aberrations (STARTRK-NG). Neuro-Oncology 24 (10), 1776–1789. 10.1093/neuonc/noac087 35395680 PMC9527518

[B13] Ding Z. Jiang M. Qian J. Gu D. Bai H. Cai M. (2024). Role of transforming growth factor-β in peripheral nerve regeneration. Neural Regen. Res. 19 (2), 380–386. 10.4103/1673-5374.377588 37488894 PMC10503632

[B14] Doebele R. C. Drilon A. Paz-Ares L. Siena S. Shaw A. T. Farago A. F. (2020). Entrectinib in patients with advanced or metastatic NTRK fusion-positive solid tumours: integrated analysis of three phase 1-2 trials. Lancet Oncol. 21 (2), 271–282. 10.1016/S1470-2045(19)30691-6 31838007 PMC7461630

[B15] Firlej V. Mathieu J. R. Gilbert C. Lemonnier L. Nakhle J. Gallou-Kabani C. (2011). Thrombospondin-1 triggers cell migration and development of advanced prostate tumors. Cancer Res. 71 (24), 7649–7658. 10.1158/0008-5472.CAN-11-0833 22037878

[B16] Frampton J. E. (2021). Entrectinib: a review in NTRK+ solid tumours and ROS1+ nsclc. Drugs 81 (6), 697–708. 10.1007/s40265-021-01503-3 33871816 PMC8149347

[B17] Fumagalli G. Monza L. Cavaletti G. Rigolio R. Meregalli C. (2020). Neuroinflammatory process involved in different preclinical models of chemotherapy-induced peripheral neuropathy. Front. Immunol. 11, 626687. 10.3389/fimmu.2020.626687 33613570 PMC7890072

[B18] Gaesser J. M. Fyffe-Maricich S. L. (2016). Intracellular signaling pathway regulation of myelination and remyelination in the CNS. Exp. Neurol. 283 (Pt B), 501–511. 10.1016/j.expneurol.2016.03.008 26957369 PMC5010983

[B19] Giarratana A. O. Prendergast C. M. Salvatore M. M. Capaccione K. M. (2024). TGF-β signaling: critical nexus of fibrogenesis and cancer. J. Transl. Med. 22 (1), 594. 10.1186/s12967-024-05411-4 38926762 PMC11201862

[B20] Giustini N. P. Oh H. Eaton K. D. (2022). Development of neuropathic arthropathy with entrectinib: case report. JTO Clin. Res. Rep. 3 (11), 100419. 10.1016/j.jtocrr.2022.100419 36340796 PMC9634017

[B21] Goyal A. Agrawal A. Verma A. Dubey N. (2023). The PI3K-AKT pathway: a plausible therapeutic target in Parkinson's disease. Exp. Mol. Pathol. 129, 104846. 10.1016/j.yexmp.2022.104846 36436571

[B22] Griffin R. J. Moloney A. Kelliher M. Johnston J. A. Ravid R. Dockery P. (2005). Activation of Akt/PKB, increased phosphorylation of Akt substrates and loss and altered distribution of Akt and PTEN are features of Alzheimer's disease pathology. J. Neurochem. 93 (1), 105–117. 10.1111/j.1471-4159.2004.02949.x 15773910

[B23] Haddadi N. Lin Y. Travis G. Simpson A. M. Nassif N. T. McGowan E. M. (2018). PTEN/PTENP1: 'Regulating the regulator of RTK-dependent PI3K/Akt signalling', new targets for cancer therapy. Mol. Cancer 17 (1), 37. 10.1186/s12943-018-0803-3 29455665 PMC5817727

[B24] Hata A. Chen Y. G. (2016). TGF-Β signaling from receptors to smads. Cold Spring Harb. Perspect. Biol. 8 (9), a022061. 10.1101/cshperspect.a022061 27449815 PMC5008074

[B25] Huang H. Kaur S. Hu Y. (2019). Lab review: molecular dissection of the signal transduction pathways associated with PTEN deletion-induced optic nerve regeneration. Restor. Neurol. Neurosci. 37 (6), 545–552. 10.3233/RNN-190949 31839616 PMC6938454

[B26] Huang T. Wang L. Liu D. Li P. Xiong H. Zhuang L. (2017). FGF7/FGFR2 signal promotes invasion and migration in human gastric cancer through upregulation of thrombospondin-1. Int. J. Oncol. 50 (5), 1501–1512. 10.3892/ijo.2017.3927 28339036 PMC5403236

[B27] Jafari M. Ghadami E. Dadkhah T. Akhavan-Niaki H. (2019). PI3k/AKT signaling pathway: erythropoiesis and beyond. J. Cell. Physiol. 234 (3), 2373–2385. 10.1002/jcp.27262 30192008

[B28] Jakowlew S. B. (2006). Transforming growth factor-beta in cancer and metastasis. Cancer Metastasis Rev. 25 (3), 435–457. 10.1007/s10555-006-9006-2 16951986

[B29] Jassim A. Rahrmann E. P. Simons B. D. Gilbertson R. J. (2023). Cancers make their own luck: theories of cancer origins. Nat. Rev. Cancer 23 (10), 710–724. 10.1038/s41568-023-00602-5 37488363

[B30] Jiang Q. Li M. Li H. Chen L. (2022). Entrectinib, a new multi-target inhibitor for cancer therapy. Biomed. Pharmacother. 150, 112974. 10.1016/j.biopha.2022.112974 35447552

[B31] Jiang T. Wang G. Liu Y. Feng L. Wang M. Liu J. (2021). Development of small-molecule tropomyosin receptor kinase (TRK) inhibitors for NTRK fusion cancers. Acta Pharm. Sin. B 11 (2), 355–372. 10.1016/j.apsb.2020.05.004 33643817 PMC7893124

[B32] Kanehisa M. Goto S. Sato Y. Kawashima M. Furumichi M. Tanabe M. (2014). Data, information, knowledge and principle: back to metabolism in KEGG. Nucleic Acids Res. 42 (Database issue), D199–D205. 10.1093/nar/gkt1076 24214961 PMC3965122

[B33] Kilic U. Caglayan A. B. Beker M. C. Gunal M. Y. Caglayan B. Yalcin E. (2017). Particular phosphorylation of PI3K/Akt on Thr308 via PDK-1 and PTEN mediates melatonin's neuroprotective activity after focal cerebral ischemia in mice. Redox Biol. 12, 657–665. 10.1016/j.redox.2017.04.006 28395173 PMC5388917

[B34] Li Z. Zou J. Chen X. (2023). In response to precision medicine: current subcellular targeting strategies for cancer therapy. Adv. Mater. 35 (21), e2209529. 10.1002/adma.202209529 36445169

[B35] Liu D. Offin M. Harnicar S. Li B. T. Drilon A. (2018). Entrectinib: an orally available, selective tyrosine kinase inhibitor for the treatment of NTRK, ROS1, and ALK fusion-positive solid tumors. Ther. Clin. Risk Manag. 14, 1247–1252. 10.2147/TCRM.S147381 30050303 PMC6055893

[B36] Love M. I. Huber W. Anders S. (2014). Moderated estimation of fold change and dispersion for RNA-seq data with DESeq2. Genome Biol. 15 (12), 550. 10.1186/s13059-014-0550-8 25516281 PMC4302049

[B37] Lu T. Li H. Zhou Y. Wei W. Ding L. Zhan Z. (2022). Neuroprotective effects of alisol A 24-acetate on cerebral ischaemia-reperfusion injury are mediated by regulating the PI3K/AKT pathway. J. Neuroinflamm. 19 (1), 37. 10.1186/s12974-022-02392-3 PMC8822821 35130910

[B38] Luo S. Li H. Mo Z. Lei J. Zhu L. Huang Y. (2019). Connectivity map identifies luteolin as a treatment option of ischemic stroke by inhibiting MMP9 and activation of the PI3K/Akt signaling pathway. Exp. Mol. Med. 51 (3), 1–11. 10.1038/s12276-019-0229-z PMC6434019 30911000

[B39] Marcus L. Donoghue M. Aungst S. Myers C. E. Helms W. S. Shen G. (2021). FDA approval summary: entrectinib for the treatment of NTRK gene fusion solid tumors. Clin. Cancer Res. 27 (4), 928–932. 10.1158/1078-0432.CCR-20-2771 32967940

[B40] Martineau C. Turcotte M. K. Otis N. Provost F. Themens L. Guay M. P. (2022). Management of adverse events related to first-generation tyrosine receptor kinase inhibitors in adults: a narrative review. Support. Care Cancer 30 (12), 10471–10482. 10.1007/s00520-022-07401-y 36326907

[B41] Meyers E. A. Kessler J. A. (2017). TGF-Β family signaling in neural and neuronal differentiation, development, and function. Cold Spring Harb. Perspect. Biol. 9 (8), a022244. 10.1101/cshperspect.a022244 28130363 PMC5538418

[B42] Mun E. J. Babiker H. M. Weinberg U. Kirson E. D. Von Hoff D. D. (2018). Tumor-treating fields: a fourth modality in cancer treatment. Clin. Cancer Res. 24 (2), 266–275. 10.1158/1078-0432.CCR-17-1117 28765323

[B43] Rai S. N. Dilnashin H. Birla H. Singh S. S. Zahra W. Rathore A. S. (2019). The role of PI3K/akt and ERK in neurodegenerative disorders. Neurotox. Res. 35 (3), 775–795. 10.1007/s12640-019-0003-y 30707354

[B44] Robinson M. D. McCarthy D. J. Smyth G. K. (2010). edgeR: a Bioconductor package for differential expression analysis of digital gene expression data. Bioinformatics 26 (1), 139–140. 10.1093/bioinformatics/btp616 19910308 PMC2796818

[B45] Staff N. P. Grisold A. Grisold W. Windebank A. J. (2017). Chemotherapy-induced peripheral neuropathy: a current review. Ann. Neurol. 81 (6), 772–781. 10.1002/ana.24951 28486769 PMC5656281

[B46] Subramanian A. Tamayo P. Mootha V. K. Mukherjee S. Ebert B. L. Gillette M. A. (2005). Gene set enrichment analysis: a knowledge-based approach for interpreting genome-wide expression profiles. Proc. Natl. Acad. Sci. U. S. A. 102 (43), 15545–15550. 10.1073/pnas.0506580102 16199517 PMC1239896

[B47] Sun J. Ge X. Wang Y. Niu L. Tang L. Pan S. (2022). USF2 knockdown downregulates THBS1 to inhibit the TGF-β signaling pathway and reduce pyroptosis in sepsis-induced acute kidney injury. Pharmacol. Res. 176, 105962. 10.1016/j.phrs.2021.105962 34756923

[B48] Trendowski M. R. El Charif O. Dinh P. C. Jr. Travis L. B. Dolan M. E. (2019). Genetic and modifiable risk factors contributing to cisplatin-induced toxicities. Clin. Cancer Res. 25 (4), 1147–1155. 10.1158/1078-0432.CCR-18-2244 30305294 PMC6377815

[B49] Tu Z. Xiao R. Xiong J. Tembo K. M. Deng X. Xiong M. (2016). CCR9 in cancer: oncogenic role and therapeutic targeting. J. Hematol. Oncol. 9, 10. 10.1186/s13045-016-0236-7 26879872 PMC4754913

[B50] Wang C. M. Cai X. L. Wen Q. P. (2016). Astaxanthin reduces isoflurane-induced neuroapoptosis via the PI3K/Akt pathway. Mol. Med. Rep. 13 (5), 4073–4078. 10.3892/mmr.2016.5035 27035665

[B51] Wang C. Y. Lin T. T. Hu L. Xu C. J. Hu F. Wan L. (2023). Neutrophil extracellular traps as a unique target in the treatment of chemotherapy-induced peripheral neuropathy. EBioMedicine 90, 104499. 10.1016/j.ebiom.2023.104499 36870200 PMC10009451

[B52] Wang Q. Shen Z. N. Zhang S. J. Sun Y. Zheng F. J. Li Y. H. (2022). Protective effects and mechanism of puerarin targeting PI3K/Akt signal pathway on neurological diseases. Front. Pharmacol. 13, 1022053. 10.3389/fphar.2022.1022053 36353499 PMC9637631

[B53] Xiao C. L. Yin W. C. Zhong Y. C. Luo J. Q. Liu L. L. Liu W. Y. (2022). The role of PI3K/Akt signalling pathway in spinal cord injury. Biomed. Pharmacother. 156, 113881. 10.1016/j.biopha.2022.113881 36272264

[B54] Yang J. Zhao Y. Zhou Y. Wei X. Wang H. Si N. (2022). Advanced nanomedicines for the regulation of cancer metabolism. Biomaterials 286, 121565. 10.1016/j.biomaterials.2022.121565 35576808

[B55] Yao L. Lu F. Koc S. Zheng Z. Wang B. Zhang S. (2023). LRRK2 Gly2019Ser mutation promotes ER stress via interacting with THBS1/TGF-β1 in Parkinson's disease. Adv. Sci. (Weinh) 10 (30), e2303711. 10.1002/advs.202303711 37672887 PMC10602550

[B56] Yu G. Wang L. G. Han Y. He Q. Y. (2012). clusterProfiler: an R package for comparing biological themes among gene clusters. OMICS 16 (5), 284–287. 10.1089/omi.2011.0118 22455463 PMC3339379

[B57] Yuan M. Huang L. L. Chen J. H. Wu J. Xu Q. (2019). The emerging treatment landscape of targeted therapy in non-small-cell lung cancer. Signal Transduct. Target. Ther. 4, 61. 10.1038/s41392-019-0099-9 31871778 PMC6914774

[B58] Zheng P. P. Li J. Kros J. M. (2018). Breakthroughs in modern cancer therapy and elusive cardiotoxicity: critical research-practice gaps, challenges, and insights. Med. Res. Rev. 38 (1), 325–376. 10.1002/med.21463 28862319 PMC5763363

