# Supplementary material for: Entrectinib can induce nerve cell damage by inhibiting PI3K-AKT and TGF-β signaling pathways
Source: Front Pharmacol. 2025 Feb 13;16:1489210. doi: 10.3389/fphar.2025.1489210 (PMC11865199; doi:10.3389/fphar.2025.1489210)
Supplement: Supplementary file 3 [file Table1.docx]

| Gene | Forward Primer | Reverse Primer |
| --- | --- | --- |
| THBS1 (human) | CCCATTCCACTCTGCCTTT | TCATACAATCGTCTCGGGTATG |
| THBS1 (mouse) | AAAGCCAAAGCGCCTATTTA | ACATGCCCTCCCTAGAGCTT |
| THBS1 (rat) | CCACTCTGCCTTACTCACAGA | CATGGCTCCTAGTGCTTTGG |
| β-actin (human) | TCACCCACACTGTGCCCATCTACGA | CAGCGGAACCGCTCATTGCCAATGG |
| β-actin (mouse) | GCTACAGCTTCACCACCACAG | GGTCTTTACGGATGTCAACGTC |
| β-actin (rat) | TCACCCACACTGTGCCCATCTATGA | CATCGGAACCGCTCATTGCCGATAG |
